# Epidemiological Characteristics of Sporadic Nosocomial COVID-19 Infections From June 2020 to June 2021 in China: An Overview of Vaccine Breakthrough Infection Events

**DOI:** 10.3389/fmed.2021.736060

**Published:** 2021-10-28

**Authors:** Zhigang He, Hongbing Xiang, Anne Manyande, Weiguo Xu, Li Fan, Boqi Xiang

**Affiliations:** ^1^Department of Anesthesiology, Tongji Hospital of Tongji Medical College, Huazhong University of Science and Technology, Wuhan, China; ^2^School of Human and Social Sciences, University of West London, London, United Kingdom; ^3^Department of Orthopedics, Tongji Hospital of Tongji Medical College, Huazhong University of Science and Technology, Wuhan, China; ^4^Department of Orthopedics, Union Hospital of Tongji Medical College, Huazhong University of Science and Technology, Wuhan, China; ^5^School of Public Health, Rutgers University, New Brunswick, NJ, United States

**Keywords:** COVID-19 disease, asymptomatic carrier, nosocomial infection, scientific protective strategy, vaccine breakthrough infection

## Abstract

The severe acute respiratory syndrome coronavirus 2 (SARS-CoV-2) pandemic has wreaked havoc on millions of people around the world. Although China quickly brought the Coronavirus disease (COVID-19) under control, there have been several sporadic outbreaks in different regions of China since June 2020. This article described the chronological nosocomial COVID-19 infection events related to several sporadic outbreaks of SARS-CoV-2 in different regions of China. We have reported epidemiological characteristics and management measures of sporadic nosocomial COVID-19 infections from June 2020 to June 2021 and specially focused on the domestic COVID-19 breakthrough infection in China, such as domestic COVID-19 breakthrough infection—a vaccinated healthcare professional working in the isolation ward of a designated COVID-19 hospital.

## Introduction

The outbreak of the Coronavirus-2019 (COVID-19) virus was first reported in Wuhan, China, in December 2019. The COVID-19 pandemic led China to quarantine the population to protect them (1). Chinese authorities decided to adopt extraordinary measures to contain and limit the spread of the severe acute respiratory syndrome coronavirus 2 (SARS-CoV-2) virus. From January 1, 2020, to April 8, 2020, >8,000 patients with COVID-19 were hospitalized, and the Chinese government imposed the Wuhan lockdown on January 23, which ended on April 8, 2020.

With the COVID-19 epidemic quickly under control in the early stages of 2020, importing the SARS-CoV-2 virus could pose great challenges to the control and prevention of nosocomial COVID-19 infection in healthcare settings. Our previous report showed the impact of the novel coronavirus SARS-CoV-2 among healthcare workers in hospitals during the early phase of the COVID-19 epidemic (2) and suggested that local authorities need to be extremely cautious and implement stringent protective measures to safeguard healthcare workers to counteract the threats brought by the pandemic (3–5). Though medical staff belongs to the susceptible population to a certain extent, hospitals have practiced cohorting in accordance with recommendations from COVID-19 infection prevention and control professional societies, which has reduced the risk of hospital-acquired COVID-19. Because healthcare workers are at the interface between hospitals and the community, where there is significant COVID-19 transmission, they may be infected by asymptomatic carriers with COVID-19 or patients with COVID-19. Furthermore, in the designated hospital admitting patients with COVID-19 or asymptomatic carriers, medical staff is highly exposed to nosocomial COVID-19 acquisition and SARS-CoV-2 transmission. Therefore, healthcare staff may play a key role in initiating or amplifying sporadic COVID-19 outbreaks in healthcare settings, such as hospitals and other care facilities.

The importing of the SARS-CoV-2 virus from overseas induced sporadic outbreaks with COVID-19 from June 2020 to June 2021 in China (6, 7). This article describes the chronological events that led to several sporadic nosocomial COVID-19 infections in different regions of China from June 2020 to June 2021, such as domestic COVID-19 breakthrough infection—a vaccinated healthcare professional working in the isolation ward of a designated COVID-19 hospital (8). Lastly, we provide an overview of the local COVID-19 outbreak induced by the B.1.617.2 (Delta) variant of the COVID-19 virus in China.

## Methods

From the beginning of June 1, 2020, every day, we prospectively focused on the COVID-19 epidemic data from the Chinese Center for Disease Control and Prevention. Once we had the new report of nosocomial COVID-19 infection in China, we tracked this epidemic and collected its epidemiological characteristics from announcements by the local Health Commission, and presented narrative research for geographical and epidemiological characteristics of nosocomial COVID-19 infection from June 2020 to June 2021.

We searched epidemiologic data published on the website of WHO, the China Center for Disease Control and Prevention, the National Health Commission, the Health Commission of Qingdao, Shenyang, Shijiazhuang, and Dalian city, Jilin, Shaanxi, and Guangdong Province from June 2020 to January 2021. Using the keywords “nosocomial infection,” “COVID-19 variant,” “SARS-CoV-2,” “B1.617.2 (Delta) lineage,” and Boolean operator “AND,” we periodically searched the published medical literature using the PubMed service maintained by the U.S. National Library of Medicine of NIH. Confirmed COVID-19 cases are defined as persons who tested positive for SARS-CoV-2 and had clinical symptoms. Asymptomatic carriers refer to persons without clinical symptoms who tested positive for SARS-CoV-2.

## Results

### Regional Distribution of Nosocomial COVID-19 Infections From June 2020 to June 2021

**Figure 1:**
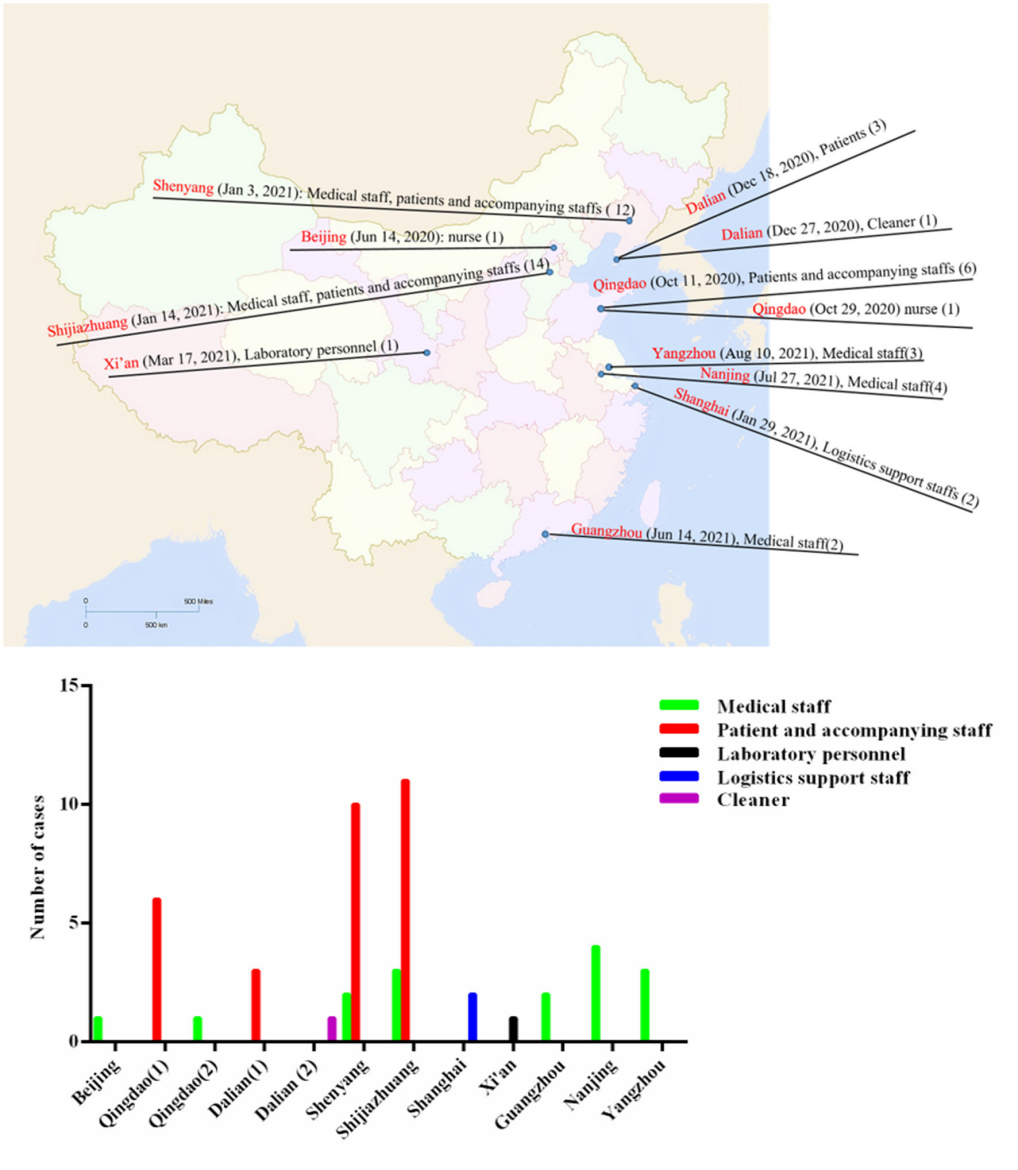
Geographical distribution of sporadic nosocomial COVID-19 infections from June 2020 to June 2021 in China. Seven cities (Dalian, Beijing, Qingdao, Shijiazhuang, Xi'an, Guangzhou, and Shanghai) reported nosocomial COVID-19 infections. Medical staff, patients, and accompanying staff (12) in Shenyang (Jan 3, 2021), patients (3) in Dalian (Dec 18, 2020), patients and accompanying staff (6) in Qingdao (Oct 11, 2020), laboratory personnel (1) in Xi'an (Mar 17, 2021), medical staff, patients and accompanying staff (14) in Shijiazhuang (Jan 14, 2021), and Medical staff (2) in Guangzhou (Jun 14, 2021) were diagnosed as infected cases.

**Figure 2:**
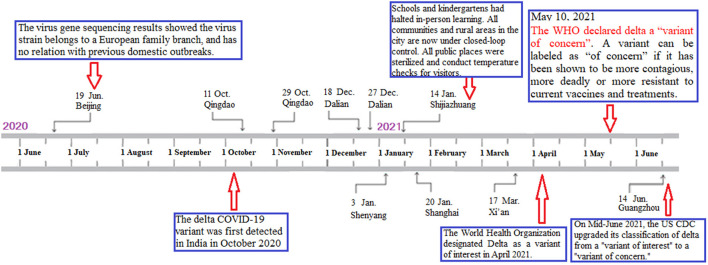
The graph's left-right axis is used as a timeline of the key events and dynamic profile of sporadic nosocomial COVID-19 infections from June 2020 to June 2021 in China. From June 2020 to June 2021, five cities in China had reported 10 series of cases with nosocomial COVID-19 infections, including 39 confirmed cases and 6 asymptomatic cases. The delta COVID-19 variant was first detected in India in October 2020. The World Health Organization designated Delta as a variant of interest in April and a variant of concern on 11 May 2021. In Mid-June 2021, the US Centers for Disease Control and Prevention upgraded its classification of the delta from a “variant of interest” to a “variant of concern”.

From June 2020 to June 2021, the regional distribution of the sporadic nosocomial COVID-19 infections is shown in Figures 1, 2 and Table 1. Most of these cities are located in coastal or airline hub areas, for example, Dalian, Qingdao, Shanghai are coastal cities, and Xi'an, Shenyang, Shijiazhuang, and Guangzhou are airline hub cities.

**Table 1:** Epidemiological characteristics of sporadic nosocomial COVID-19 infections.

**Date**	**City**	**Number of hospital/cases**	**Hospital type**	**Profession**	**Classification of infection**	**Route of infection**	**Database**
19 June 2020	Beijing	1/1	Designated hospital	Nurse	Community-acquired infection	Family member exposure with COVID-19	Link 1
11 October 2020	Qingdao	1/12	Designated hospital	Patients and accompanying staff	Hospital-acquired infection	Nosocomial infection	Link 2
29 October 2020	Qingdao	1/1	Designated hospital	Nurse	Hospital-acquired infection	Workplace accidental exposure to COVID-19	Link 3
18 December 2020	Dalian	1/3	Non-designated hospital	Patients	Hospital-acquired infection	Nosocomial infection	Link 4
27 December 2020	Dalian	1/1	Non-designated hospital	Cleaner	Community-acquired infection	Cold-chain environment-to-human transmission	Link 5
3 January 2021	Shenyang	1/12	Non-designated hospital	Medical staff, patients and accompanying staff	Hospital-acquired infection	Nosocomial infection	Link 6
14 January 2021	Shijiazhuang	2/14	Non-designated hospital	Medical staff, patients and accompanying staff	Hospital-acquired infection	Nosocomial infection	Link 7
20 January 2021	Shanghai	2/2	Non-designated hospital	Logistics support staff	Community-acquired infection	Imported from overseas	Link 8
17 March 2021	Xi'an	1/1	Designated hospital	Laboratorian	Hospital-acquired infection	Mild breakthrough infection	Link 9
14 June 2021	Guangzhou	1/2	Designated hospital	Laboratorian and emergency doctor	Hospital-acquired infection	Nosocomial infection	Link 10
27 July 2021	Nanjing	1/4	Non-designated hospital	Accompanying staff	Hospital-acquired infection	Nosocomial infection	Link 11
10 August 2021	Yangzhou	1/3	Non-designated hospital	Doctor	Hospital-acquired infection	Nosocomial infection	Link 12

*Designated hospital: a hospital designated for treatment of imported COVID-19 cases.*

*Link 1: https://finance.sina.com.cn/china/gncj/2020-06-19/doc-iircuyvi9385332.shtml?r=9&tj=none&tr=9.*

*Link 2: https://www.zhihu.com/question/425220159.*

*Link 3: https://new.qq.com/omn/20201030/20201030A05GXU00.html.*

*Link 4: http://www.xinhuanet.com/politics/2021-01/06/c_1126953634.htm.*

*Link 5: https://new.qq.com/omn/20201229/20201229A0259M00.html.*

*Link 6: https://new.qq.com/omn/20210111/20210111A0DFD500.html.*

*Link 7: https://news.sina.com.cn/c/2021-01-20/doc-ikftpnnx9631142.shtml.*

*Link 8: https://new.qq.com/omn/20210131/20210131A0071200.html.*

*Link 9: https://news.sina.com.cn/c/2021-03-18/doc-ikkntiam4831560.shtml.*

*Link 10: https://news.sina.com.cn/c/2021-06-14/doc-ikqcfnca0979774.shtml.*

*Link 11: http://med.china.com.cn/content/pid/281087/tid/1026.*

*Link 12: http://news.hexun.com/2021-08-18/204179459.html.*

### Occupational Distribution of Nosocomial COVID-19 Infections From June 2020 to June 2021

From June 2020 to June 2021, six cities in China had reported over 45 cases with nosocomial COVID-19 infections, i.e., 39 confirmed cases and 6 asymptomatic cases (Table 2). Among them, nursing staff (3), doctors (6), patients (12), accompanying staff (22), and laboratorian (2) were diagnosed. The route of these nosocomial infections mainly included workplace accidental exposure to COVID-19, cross-infection among healthcare workers, patients, and accompanying staff.

**Table 2:** Epidemiological characteristics of sporadic nosocomial COVID-19 infections.

**Date**	**City**	**Confirmed/** **asymptomatic cases**	**How to discover the source of infection**	**Virus type**	**Database**
19 June 2020	Beijing	1/0	Nucleic acid testing for close contacts of people with infection cases	One ancestral virus strain (XFDM strain) (9)	Link 1
11 October 2020	Qingdao	6/6	Regular screening for medical staff	Imported virus strain Lineage B.1.1 (10)	Link 2
29 October 2020	Qingdao	1/0	Regular screening for medical staff	Imported virus strain Lineage B.1.1 (10)	Link 3
18 December 2020	Dalian	3/0	Regular screening for medical staff	European family branch 1 of the L genotype	Link 4
27 December 2020	Dalian	1/0	Regular screening for medical staff	European family branch 1 of the L genotype	Link 5
3 January 2021	Shenyang	12/0	Regular screening for medical staff	Imported virus strain	Link 6
14 January 2021	Shijiazhuang	14/0	Regular screening for medical staff	the strain imported from Europe (11)	Link 7
20 January 2021	Shanghai	2/0	Nucleic acid testing for close contacts of people with infection cases	Imported virus strain	Link 8
17 March 2021	Xi'an	1/0	Regular screening for medical staff	Imported virus strain B.1.1.7 (8)	Link 9
14 June 2021	Guangzhou	2/0	Regular screening for medical staff	Imported virus strain B1.617.2 (Delta) Lineage	Link 10
27 July 2021	Nanjing	4/0	Regular screening for medical staff	Imported virus strain B1.617.2 (Delta) Lineage	Link 11
10 August 2021	Yangzhou	3/0	Regular screening for medical staff	Imported virus strain B1.617.2 (Delta) Lineage	Link 12

*Delta Lineage: the highly contagious Covid-19 variant first identified in India.*

*Link 1: http://weekly.chinacdc.cn/en/article/doi/10.46234/ccdcw2020.246.*

*Link 2: http://weekly.chinacdc.cn/en/article/doi/10.46234/ccdcw2020.224.*

*Link 3: https://new.qq.com/omn/20201030/20201030A05GXU00.html.*

*Link 4: http://www.xinhuanet.com/politics/2021-01/06/c_1126953634.htm.*

*Link 5: https://new.qq.com/omn/20201229/20201229A0259M00.html.*

*Link 6: https://new.qq.com/omn/20210111/20210111A0DFD500.html.*

*Link 7: http://weekly.chinacdc.cn/en/article/doi/10.46234/ccdcw2021.006.*

*Link 8: https://new.qq.com/omn/20210131/20210131A0071200.html.*

*Link 9: http://weekly.chinacdc.cn/en/article/doi/10.46234/ccdcw2021.094.*

*Link 10: https://news.sina.com.cn/c/2021-06-14/doc-ikqcfnca0979774.shtml.*

*Link 11: http://med.china.com.cn/content/pid/281087/tid/1026.*

*Link 12: http://news.hexun.com/2021-08-18/204179459.html.*

### SARS-CoV-2 Gene Sequencing Results of Nosocomial COVID-19 Infections From June 2020 to June 2021

Severe acute respiratory syndrome coronavirus 2 gene sequencing is crucial work for nosocomial COVID-19 infections in new sporadic outbreak regions. The virus strain of nosocomial COVID-19 infection found in Qingdao city is the virus strain lineage B.1.1 imported from overseas (Table 2). Gene sequencing results showed that the coronavirus found in Dalian, Shenyang, and Shijiazhuang cities is similar to the strain imported from Europe. The virus strain found in Xi'an city is a COVID-19 variant B.1.1.7 lineage, and the SARS-CoV-2 strain found in Guangzhou city is the imported virus strain B1.617.2 (Delta) lineage (Table 2).

A report from Fang et al. (12) showed that the strains associated with a specific outbreak in Dalian City were as follows: LNDL-BHQ-0722-Y_S12_L001_R1_001, LNDL-SFL-0722-Y_S9_L001_R1_001, LNDL-WY-0722-Y_S11_L001_R1_001, and LNDL-XY-0722-Y_S10_L001_R1_001, and the parent strain from Wuhan was NC_045512.2_Severe_acute_respiratory_syndrome_coronavirus_2_isolate_Wuhan-Hu-1_complete_genome. After accessing the public database GISAID and GenBank, three Russian strains detected in July were found to share the 10 variation sites with the two Hebei strains (GISAID IDs: EPI_ISL_596266, EPI_ISL_569792, and EPI_ISL_569793) (13). The evidence indicates that the Shijiazhuang strains may have originated from this Russian strains (13).

## Discussion

This study provides us with an inspiring vision regarding the current COVID-19 pandemic. The main findings of sporadic nosocomial COVID-19 infections from June 2020 to June 2021 are listed below: (1) the importance of rolling out an overall nucleic acid test campaign to all staff in healthcare settings is a crucial part of the COVID-19 surveillance; (2) the case of coronavirus transported onto the workplace warns us that the current hospital disinfection concept urgently requires updating, especially in designated hospitals, and there is an urgent demand of the management of staff accompanying patients in non-designated hospitals; (3) The phenomenon about COVID-19 vaccine breakthrough infection deserves our great attention.

For healthcare workers, patients/accompanying staff, and cleaners, their COVID-19 infection may be from a hospital (hospital-acquired infection, also nosocomial infection) or community (community-acquired infection). Distinguishing nosocomial infection and community-acquired infection is an important base to control the epidemic in nosocomial COVID-19 infections. Because healthcare workers are at the interface between hospitals and the community, where there is significant COVID-19 transmission, they may be infected by asymptomatic carriers from the community with COVID-19 or patients with COVID-19. These healthcare workers (infected in the community) do not belong to nosocomial infection (also called hospital-acquired infection). For example, in Figure 1, the infected cases (such as healthcare workers, cleaners, and so on) in Beijing, Dalian (December 27, 2020), and Shanghai were community-acquired infections, not nosocomial infections. For nosocomial infection of healthcare workers, patients/accompanying staff, and cleaner, the effective control measures taken by the local government must include: (1) these hospitals imposed lockdown, especially in the designated COVID-19 hospital; (2) rolling out an overall nucleic acid test campaign to all staff is key in healthcare settings.

Among nine sporadic nosocomial COVID-19 infections, our results showed that eight series cases were found by rolling out overall a nucleic acid test campaign to all hospital staff, suggesting the importance of regular screening for all staff in healthcare settings. As part of efforts to control the COVID-19 infections, one of the effective prevention measures taken by local authorities is that all hospital personnel labeled a key population is closely monitored (7). Whether the epidemic in nosocomial COVID-19 infections can be brought under control depends on how many new infections emerge of their close contacts and secondary close contacts in the next 2 weeks. If these hospitals imposed lockdown as the effective control measures taken by the local government, then ideally, new confirmed cases with COVID-19 infections will see a downward trend within 2 weeks. Cases, such as hospital-acquired infection in the designated hospital of Qingdao city, are a warning that workplace storage and transport could be a hotbed for the coronavirus or other pathogens. There is an urgent demand for workplace disinfection to protect the health safety of medical staff, patients, and accompanying staff in the current pandemic or into another worse outbreak.

Our data also showed that a vaccinated healthcare professional who received inactivated vaccine was infected with COVID-19 while working in the isolation ward of a designated COVID-19 hospital, and the coronavirus strain was determined to be the imported COVID-19 variant strain B.1.1.7 (8), suggesting that there exists the domestic COVID-19 vaccine breakthrough infection in China, and this phenomenon deserves our serious attention. Vaccination is well-known to be key to stopping the virus from circulating and more variants from popping up (14–16). The vaccine breakthrough infection case was defined as an individual with positive SARS-CoV-2 nucleic acid amplification tested after receiving at least one dose of a SARS-CoV-2 vaccine (17–19). Jacobson et al. (20) addressed post-vaccination SARS-CoV-2 infections and the incidence of the B.1.427/B.1.429 variant among healthcare personnel at a northern California academic medical center. Hacisuleyman et al. (21) reported two women with vaccine breakthrough infection in a cohort of 417 persons who had received the second dose of BNT162b2 (Pfizer–BioNTech, New York, NY, USA) or mRNA-1273 (Moderna, Cambridge, MA, USA) vaccine at least 2 weeks previously, and the viral sequencing showed that they were infected with the new variant virus, namely, E484K in one woman and three mutations (T95I, del142–144, and D614G) in both. These observations revealed a potential risk of COVID infection with the variant virus after successful vaccination.

Vaccine breakthrough cases with SARS-CoV-2 were reported in many countries (22–24). An analysis from the vaccination campaign of Israel showed that COVID-19-related hospitalizations, severe disease, and death were reduced in infected cases with SARS-CoV-2 after vaccination, such as symptomatic and asymptomatic infections (24). Antonelli et al. (25) identified risk factors for post-vaccination SARS-CoV-2 infection and describe the characteristics of post-vaccination illness and found that Almost all symptoms were reported less frequently in infected vaccinated individuals than in infected unvaccinated individuals, and vaccinated participants were more likely to be completely asymptomatic, especially if they were 60 years or older. Our vaccine breakthrough case from Xi'an supported vaccine effectiveness and cautioned around relaxing physical distancing and other personal protective measures in the post-vaccination era.

Severe acute respiratory syndrome coronavirus 2 virus delta variants have drawn worldwide attention. The WHO proposed three labels for global SARS-CoV-2 variants, namely, a variant of concern (VOC), variant of interest (VOI), and variant under monitoring, to be used alongside the scientific nomenclature in communications about variants to the public (26). The B.1.617 variant of the COVID-19 virus has been called a triple mutant variant since it splits into three lineages, namely, the B.1.617.1 (Kappa) variant, the B.1.617.2 (Delta) variant, and the B.1.617.3 variant (27). The delta COVID-19 variant, which was first detected in India in October 2020, had been reported in more than 80 countries on June 20, 2021 (28). The WHO declared the delta variant a “VOC” on May 10, 2021. In mid-June 2021, the US Centers for Disease Control and Prevention upgraded its classification of the delta from a “VOI” to a “VOC” (29). Our results showed that two medical staff (one laboratorian and one emergency doctor), infected with COVID-19 in the designated hospital, occurred due to workplace accidental exposure to COVID-19, and the coronavirus strain was determined to be the imported B.1.617.2 (Delta) variant.

To conclude, our findings add to the accumulating evidence regarding the importance of regular screening for all staff in healthcare settings. Furthermore, our study highlights the need for an update of the current hospital disinfection procedures in designated hospitals to prevent the nosocomial spread of SARS-CoV-2 infection. Finally, the epidemiological exposure of vaccinated medical staff should draw concern to minimize the impact of a new outbreak induced by virus mutants.

## Data Availability Statement

The original contributions presented in the study are included in the article/supplementary material, further inquiries can be directed to the corresponding author/s.

## Author Contributions

All the authors contributed to the paper's presented methodology, conceptualization, data analysis, and paper writing.

## Funding

This work was funded by the National Natural Science Foundation of China (81873467, 81670240, and 81271766).

## Conflict of Interest

The authors declare that the research was conducted in the absence of any commercial or financial relationships that could be construed as a potential conflict of interest.

## Publisher's Note

All claims expressed in this article are solely those of the authors and do not necessarily represent those of their affiliated organizations, or those of the publisher, the editors and the reviewers. Any product that may be evaluated in this article, or claim that may be made by its manufacturer, is not guaranteed or endorsed by the publisher.
